# English Veterinary Degrees

**Published:** 1882-01

**Authors:** 


English Veterinary Degrees.—The British Parliament recently passed a very important measure, looking to the protection of the veterinary profession. This act will have the effect of raising to the rank of other liberal professions that of the veterinarian, which has so long labored under the disadvantage of an unprotected title. It also enables those desiring treatment for animals to distinguish between a qualified surgeon and one who assumes the title without any education fitting him therefor, as all the students of the various medical colleges in the United Kingdom are required to pass a rigid examination by the Council of the Royal College of Veterinary Surgeons, and any person assuming the title of veterinary surgeon will be guilty of a penal offence, and liable to a fine of twenty pounds, with a view to treating justly and with consideration, practitioners of veterinary medicine who, by their skill and experience, have established a reputation. The act gives to such persons, if they have been practicing continuously for five years, the right to enroll themselves in the register of the College as "Existing Practitioners." Another very important feature of the act is that preventing any but qualified practitioners to recover in a court of law any fee or charge for performing any veterinary operation or for giving any veterinary attendance or advice.

